# The art and science of priority-setting: assessing the value of Public Health England’s Prioritization Framework

**DOI:** 10.1093/pubmed/fdaa016

**Published:** 2020-02-06

**Authors:** G Maniatopoulos, D J Hunter, J Gray

**Affiliations:** 1 Institute of Health and Society, Newcastle University, Newcastle upon Tyne, UK; 2 Health and Life Sciences, Northumbria University, Newcastle upon Tyne, UK

**Keywords:** public health, priority-setting, Public Health England

## Abstract

**Background:**

Findings are presented from the evaluation of Public Health England’s (PHE) Prioritization Framework (PF) aimed to assist local authority commissioners with their public health investment and disinvestment decisions. The study explored the take up of the PF in three early adopter local authority settings.

**Methods:**

Semi-structured interviews (*n* = 30) across three local authorities supplemented by participant observation of workshops.

**Results:**

Participants acknowledged that the PF provided a systematic means of guiding priority-setting and one that encouraged transparency over investment and disinvestment decisions. The role performed by PHE and its regional teams in facilitating the process was especially welcomed and considered critical to the adoption process. However, uptake of the PF required a significant investment of time and commitment from public health teams at a time when resources were stretched. The impact of the political environment in the local government was a major factor determining the likely uptake of the PF. Ensuring committed leadership and engagement from senior politicians and officers was regarded as critical to success.

**Conclusions:**

The study assessed the value and impact of PHE’s PF tool in three early adopter local authorities. Further research could explore the value of the tool in aiding investment and disinvestment decisions and its impact on spending.

## Background

Local authority public health teams are required to make decisions about how best to prioritize the funds available to them in order to achieve the maximum health gain for their local communities.^1^ The investment and disinvestment decisions they make are even more critical at a time of shrinking budgets.^2^ The Health and Social Care Act^3^ (2012) in England established the new statutory role of public health within local authorities.^4–7^ As a result, the local government in England is responsible for providing public health services funded via a ring-fenced grant allocated by the central government. Each local authority has a public health team of varying size headed up by a director of public health (DPH). Public health teams are multidisciplinary in nature. Working with other local authority service directors, elected members and the chief executive, these teams determine public health strategy and the priorities accorded different programmes. Understanding how public health priorities are determined in the local government, and how priority-setting tools might support decision-making, remains unclear.^8^

Over the years a number of priority-setting tools have been developed to facilitate decision-making processes in public health.^9^ Most recently, Public Health England (PHE) has developed a Prioritization Framework (PF).^8^ Launched in March 2018, the PF is intended to assist local government public health commissioners and decision-makers with their budget allocations to support public health interventions. In particular, the PF is designed to help identify areas of service provision that might benefit from investment or, conversely, disinvestment. Drawing upon the principles of multi-criteria decision analysis,^10^ the PF aims to support strategic decision-making in many different public health circumstances and contexts such as multi-agency working. The PF facilitates the evaluation of the programmes that can offer the best value, the current states of the programmes, the budgets and how they are currently allocated across programmes and how easy it could be for the programmes to change and improve. The process allows public health programmes to be scored on the potential state, current state and the programme budgets while, at the same time, considering what is achievable. The purpose of this is to be able to make informed recommendations on whether to increase, decrease or maintain budget spending in each public health programme.

Against this background, this paper presents the findings from a National Institute for Health Research (NIHR) School for Public Health Research (SPHR) study aimed at assessing the value and impact of the tool in three early adopter local authorities.^11^ It follows on from an earlier SPHR project aimed at exploring methods to support priority-setting efforts to improve population health and address health inequalities.^8–9^ The paper explores the likely acceptability and utility of PHE’s PF tool and the perceived opportunities and challenges for decision-making.

The paper is structured as follows. First, the methods adopted to collect data are described. Second, findings are presented in regard to participants’ views on the PF tool and the opportunities and challenges in using it to inform their decision-making. Finally, issues common across all three local authorities are considered, identifying any emerging lessons with a view to informing the future design and adoption of the PF tool.

**Table 1:** List of interviewees

*Site*	*No. of interviews*	*Interviewees*
Site 1	8	PH Consultant x 2
		Portfolio Lead x 2
		PH Consultant/Project Lead
		Senior PH Consultant/Specialist
		Strategic Commissioning Manager
		Director of PH
Site 2	8	Deputy Director of PH
		Development and Implementation Lead
		Director of PH
		PH Manager
		Senior PH Information Analyst
		PH Lead x 2
		Councillor/Elected Member
Site 3	11	Head of PH Business Programmes
		PH Consultant x 3
		PH Director
		Senior Finance Business Partner
		Councillor/Elected Member x 5
PHE	3	PHE Regional Manager x 3
Total	30	

## Methods

Data were collected through semi-structured interviews (30 in total; see Table 1 for a list of roles) conducted between January and July 2018 with key informants in three early adopter local authority sites, supplemented by participant observation of PF workshops. Members of the research team evaluating the PF had no previous involvement with the development of the PF tool, although the tool was informed by the findings from the earlier SPHR funded research mentioned above. Neither did the research team have any prior involvement with any of the respondents located in the early adopter sites. The workshops took place within each LA’s offices and involved the participation of a wide range of stakeholders from public health and other departments. During the workshops public health teams within each site actively engaged with the PF tool to inform evidence-based spending decisions across a number of public health programmes. In this context, the purpose of the workshops was the real-time implementation and testing of the PF tool to support decision-making in public health spending. In one site, elected members also participated in the workshops. All workshops were chaired by the DPH or a senior public health consultant with the support of a PHE regional manager and often involved lengthy discussions among stakeholders. There was a degree of uncertainty and confusion among some of the stakeholders about the contribution of the tool to the prioritization process. Often these meetings would be dominated by certain public health consultants, but the chairs of the workshops would attempt to counteract this through soliciting the views of others. A brief summary of each local authority health profile is provided in Table 2.

**Table 2:** Local authority health profiles

*Site*	*Geographical status*	*Population (2016)*	*Health in summary*	*Life expectancy*	*Health inequalities*
1	Urban	556 000	Worse than the England average	Lower than the average	Life expectancy is 7.7 years lower for men and 7.1 years lower for women in the most deprived areas of the county than in the least deprived areas
2	Rural	338 000	Better than the England average	Higher than the average	Life expectancy is 6.9 years lower for men and 3.8 years lower for women in the most deprived areas of the county than in the least deprived areas
3	Urban	645 000	Better than the England average	Higher than the average	Life expectancy is 6.3 years lower for men and 5.0 years lower for women in the most deprived areas of the County than in the least deprived areas

Source: https://fingertips.phe.org.uk/profile/health-profiles.

Interviewees were purposively selected according to their role and involvement in the PHE PF project. Interviews explored respondents’ perceptions and experiences of using the PF tool and identified any barriers and facilitators to its adoption. Participants were provided with information sheets in advance and consent forms signed prior to the start of the interviews. A topic guide was developed to guide the interviews, but the emphasis was on encouraging participants to discuss and reflect upon their own perspectives and experiences. Interviews took ~30–60 min to complete. Interviews ceased once it became clear that no new themes were emerging from the data. With the permission of interviewees, all interviews were audio-recorded and transcribed. Those agreeing to be interviewed were able to withdraw at any time during the study although none did. A positive ethical opinion was obtained from Newcastle University Faculty of Medical Sciences Ethics Committee (ref: 1443/2629/2018). NHS Research Ethics approval was not required for this study. Research approval was gained in each site.

Transcribed interview data and fieldwork notes were analysed using thematic analysis to generate category systems and repeated themes.^12^ Drawing upon an interpretative approach, themes were developed iteratively and inductively, breaking down and reassembling the data through a coding process. To assure confidentiality, all those taking part in the study have been anonymized.

## Results

All three LAs, albeit to varying degrees, completed the process of using the PF and made recommendations to change budget allocations. By utilizing the tool, the public health teams engaged with a process that facilitated how to get the best value for money from the public health budget. Specifically, teams considered which programmes could offer the greatest value in the future (potential), the current state of programmes that were being delivered and how the budget was currently divided across programmes (current state) and, finally, how easy it was for programmes to change (feasibility).

### Opportunities

#### Encouraging transparency over investment/disinvestment

Across all three sites, it was acknowledged that the adoption of the PF tool provided a systematic framework to structure and guide prioritization decisions. Reflecting the ongoing financial pressures on public health budgets, and on local government spending more generally, our respondents acknowledged that the adoption of the tool could encourage transparency over investment/disinvestment decision-making in public health spending. Such a context proved receptive to adopting the PF and exploring its potential utility and value. In keeping with the uncertainty surrounding the future of public health budgets, interviewees reflected on the potential opportunities of the PF to respond to government pressures to produce efficiency savings and the need to improve the quality of services for the local population.

*Austerity, reducing budgets, makes the use of these tools even more important because as the money goes down you have got to make increasingly difficult choices. You cannot do what you have done in the past when budgets were more generous, therefore something has to go. We may decide we need to do more things rather than cut things or the impact of austerity across the wider population would mean we might need to recalibrate what we do and refocus on more vulnerable populations.* (PH Consultant, Site 1)

Although respondents across all sites shared a view that public health teams had good relationships with the rest of the local authority, it was felt that the adoption of the PF tool could help raise the profile of public health teams and also contribute to the wider understanding of the prioritization process across the council.

#### Improving collaboration and shared learning

Across all three sites, it was acknowledged that the adoption of the PF tool provided a platform for greater collaboration and shared learning between different public health professionals with the potential that this offers to improve investment/disinvestment decisions in public health spending. In particular, emphasis was given to the participatory nature of the PF tool which it was felt encouraged and enabled collective learning. Interviewees claimed that the tool could act as a mirror on, and a window into, a stakeholder’s perspective, thus facilitating the nurturing of a consensus over investment/disinvestment decisions.

*…in the past, if we have been guilty of not, perhaps, collectively sitting down together and going through financial aspects, through some of the key things that this tool potentially could bring out. Because it was very challenging for people. I do not think people had been through that environment before, actually systematically going through a tool, and potentially thinking how this could affect their commissioning… Because everyone’s got different projects, and I think sometimes people just consider their own, and do not consider knock-on effects...* (Senior PH Information Analyst, Site 2)

Reflecting on the opportunities provided by the PF tool, some interviewees supported its wider adoption across the local authority to inform budget decisions in other areas. One of our study sites subsequently employed the PF to inform spending decisions in social care.

#### Promoting effective relationships and communications

There was evidence from our first-hand observations of the workshops that the adoption of the PF tool facilitated conversations across different stakeholders which was considered to be essential if public health teams are to overcome the traditional silos in which they operate. Moreover, it was recognized by all our interviewees that the adoption of the tool could improve understanding of public health spending and also contribute to reducing the level of protectionism across programme area budgets.

*Instead of just having competing priorities, because all the things that they do are important, you can have a more logical discussion about where we are spending more or less and for what reason and what outcomes we are having and have a more informed discussion, I suppose, rather than just pinions or just looking where we spent less than we budgeted last year, for example…* (Senior Finance Business Partner, Site 3)

Although each site experienced a variety of types of engagement by key stakeholders, there was much praise for the role of the external facilitator as a ‘process owner’. Across all sites, PHE played an active role in the organization and delivery of the workshops, and its input was considered critical to the adoption of the tool.

### Challenges

#### Uncertainty around the future of PH budget

Despite these opportunities arising from the PF, our findings demonstrated that significant financial tensions and limited availability of resources, uncertainty around policy and fundamental questions about the future of the ring-fenced public health budget could hinder the adoption of the PF tool and make decision-makers wary of its purpose and impact.

*I suppose one of the other key things that we are aware of, being in the climate we have been in, of reduction on reduction on reduction, and the coming of business rates, it was looked at suspiciously to start with. What was it going to be used for? Yes, there was certainly an element of suspicion there. Rightly so, to be honest, because your timing for it is not the best in the world. And that’s human nature. Let us be honest.* (Development and Implementation Lead, Site 2)

In keeping with government pressures for efficiency savings, respondents stressed the difficulty in setting priorities for allocating a limited pool of resources

*The continuous squeeze has meant a lot of change. If anything, it would be change exhaustion. You get to a point where people say, “I’ve had enough.” Again, I suppose you might say it’s how it’s introduced. That relates to the flexibility and the ease and the time effective of the tool. It’s quite important because there has been so much change, “This is another new thing.” “We want you to spend x number of days on doing it.” It’s not fundamental I think…* (PH Consultant, Site 3)

#### The political context of local government

Many interviewees highlighted the effect of the political environment on prioritization decision-making. In particular, it was felt that the political context in which prioritization occurs (i.e. local government) could hinder the adoption of the PF tool.

*I think from a political perspective, there will always be a political element that will need to be overlaying with any prioritisation process in terms of what’s important politically. How do you balance that with what’s come out of the tool?* (Director of PH, Site 1)

It was recognized that any decision-making approach will need to take into account the local political context and organizational agenda, acknowledging that elected members will take the final decision.

*The decisions are made by the politicians. People pay their taxes and active members of the Cabinet sign off the contracts. So, that contract needs to have a decision record with it. The decision record would have a business case in it… In terms of who decides the balance between what you spend on one thing and the other, it’s not the council officer’s recommendations, it’s the Cabinet, the politicians who decide what the priorities are within the council.* (Deputy Director of PH, Site 2)

In this context, it was acknowledged that ensuring support and committed leadership from senior management was a key enabler to success. In particular, our respondents felt that elected members’ buy-in at an early stage could facilitate the adoption process and avoid problems of ownership at a later stage.

#### Limited time and resources

From an organizational perspective, it was acknowledged that the adoption of the PF tool requires a significant investment of time and commitment from public health teams. In particular, concerns were raised over the time required to populate the evidence templates by programme area leads.

*It’s just the amount of time it takes and the number of sessions it needs to pull everybody to get in to do it, and it’s one of these things, again, it’s about investing in time to do that. So I think an organisation has to be invested in doing this to take it forward…* (PH Consultant 1, Site 3)

Moreover, limited capacity among public health teams and challenges in getting the right people together at the same time were thought to be a major barrier to the effective adoption of the PF.

*…I think that capacity is one issue…because, the way that the tool is designed is you get somebody at a fairly senior level, sort of a consultant level, to be able to deliver… To be in charge of running the tool, and chasing people up, and doing their things… So, I think if we’d had more capacity there would’ve been more time for briefing, more time for understanding. Well, we were never going to do that anyway. We’d never have that capacity.* (Director of PH, Site 2)

For some respondents, uneven attendance at workshops could hinder the wider ownership and therefore successful adoption of the new tool.

*The downside is the team that started the process is not the team that’s going to complete the process. We do not have the luxury of doing it sequentially, so we have got to make the best we can.* (PH Consultant, Site 1)

Some respondents suggested that having pre-populated evidence templates provided by PHE as well as ensuring continuity of participants could improve the appeal and adoption of the tool.

#### Availability of evidence

In terms of the prioritization exercise, our respondents acknowledged difficulties in relation to the different sources and types of evidence that might be used by various stakeholders involved in making decisions. In addition, there was a general perception that limited availability of information and evidence in some areas (such as for mental health services) could hinder adoption of the tool.

*…because there is more of a history of working in some areas and more of an evidence base of working in some areas…it is difficult because if there’s NICE guidance and there’s this and there’s that, somehow has a more weighted evidence than the fact that I’ve been working with this particular community and they have told me these things… and there is a hierarchy to the evidence base, but I think sometimes that can get in the way of a more community-based approach.* (PH Consultant, Site 1)

Of particular concern among all our respondents was the lack of national indicators in certain areas of public health and an absence of qualitative evidence to inform prioritization decision-making.

*I think that some people did not essentially buy into it, but maybe because of those reasons, and wanting a more rounded sort of… I mean, we have got some people with qualitative research backgrounds who feel that they want a bit more of a nuanced approach to things, taking a whole variety of different views into account in a different methodological way…* (Director of PH, Site 2)

Moreover, across all three sites, it was acknowledged that there was a tension between national evidence and policy directives versus local needs and priorities.

*I mean obviously we have to abide by national guidelines, national standards and things within any of the services that we commission, but then we need to have the case that the need is here within the local area and that we are appropriately meeting the needs of the residents…* (PH Consultant, Site 3)

#### Stakeholder acceptability

Across all three sites, there was evidence from our observations that the workshops were favourably received and participants felt they were helpful, informative and well-structured. However, some respondents believed the PF tool was too linear, mechanistic and deterministic in its design and thus risked failing to address the dynamic, complex and multifaceted nature of the prioritization process in public health.

*I also think the thing that we have always got to be careful of in public health is not being labelled as being too academic and too technical and we do, to a certain extent, get a little bit of that label in local government. So I think those are some of the barriers and I think, for lots of people, they might find it just a bit too technical and a bit too long-winded.* (Senior PH Consultant/Specialist, Site 1)

*I think some people did not buy into the model on academic or philosophical grounds. The principles, seeing the model as too positivist, and as being a mechanistic way to determine prioritisation which maybe did not take enough other factors into account.* (Director of PH, Site 2)

There were recognized problems around assisting both elected members and staff to understand the underlying principles of the tool. Our respondents suggested that a lack of understanding of the PF tool’s contribution to the prioritization decision-making process could hinder its adoption.

*Well, it was always introduced as a pilot as such, but it wasn’t clear throughout the workshops whether or not it was going to be until, I think, we probably got to the later workshops that it was considered more of a strategic tool to inform or to consider as part of wider evidence. Budget setting in the future. So the first couple of workshops I think we are at a little bit muddled and maybe some of the more senior managers had a clearer idea as to what they thought we were going to use with the tool, but that maybe wasn’t clear to some of the commissioners and leads who were actually populating and spending time gathering the evidence.* (PH Lead 1, Site 2)

In addressing these challenges, it was acknowledged that achieving a shared understanding of the benefits of the PF tool and improving relevant stakeholder engagement could determine successful adoption. In this regard, respondents suggested that framing the value of the PF tool in the context of the prioritization process is as important as ensuring stakeholders’ engagement. Some respondents proposed the provision of supporting documents and an instructional video in order to facilitate this process.

## Discussion

While the context for each early adopter local authority site is inevitably specific and distinct and must always be taken into account when adopting the PF or a variant of it, we identified a set of common issues and themes shaping the adoption of the PF tool across the three study sites. Overall, the PF was welcomed by all three local authorities and was regarded as a useful platform for incorporating costs and benefits into decision-making and framework for evidence-informed decision-making and taking into account different types of evidence (e.g. tacit/experiential, scientific). It was acknowledged that the PF created the space for decision-makers to come together and, in an open and transparent way, seek to identify those areas of public health where investment would most benefit local communities and improve their health. Conversely, the PF identified areas where there was potential to disinvest in order to enable those resources to be put to better use in other public health areas. There was evidence from our observations of the workshops that the adoption of the PF tool facilitated conversations across different stakeholders. In this context, emphasis was given to the participatory nature of the tool which it was felt encouraged and enabled collaboration and shared learning between different public health professionals. The role performed by PHE in facilitating the process in the three sites was especially welcomed and considered to be critical to the adoption of the PF. Some interviewees supported the wider adoption of the PF across the local authority to inform budget decisions in other areas. However, it was acknowledged that the complexity surrounding the political context and organizational particularities of local government pose the greatest challenge to the scale-up and spread of the PF tool.

Despite these opportunities arising from the PF, our findings demonstrated that there were aspects of the PF which required attention and modification in order to render it even more useful and attractive to local authorities. Using the tool required considerable investment of time by public health teams, and it was thought the process could be speeded up if PHE could assist in providing the evidence underpinning decisions to invest or disinvest. Moreover, in line with previous evidence exploring challenges to public health decision-making, the impact of the political environment in local government was a major factor determining the likely uptake of the PF.^13^ Ensuring committed leadership and engagement from senior politicians as well as officers was regarded as critical to success. At the same time, significant financial pressures for efficiency savings and fundamental questions about the future of the ring-fenced public health budget could hinder the adoption of the PF tool and make decision-makers wary of its purpose and impact. In addition, there was a general perception that limited availability of information and difficulties in relation to the different sources and types of evidence in some areas of public health such as mental health services could adversely affect the take-up of the PF tool. This is consistent with previous studies which explore challenges related to the use evidence to inform local public health decision-making.^14–16^

### What is already known on this topic

Priority-setting tools play a key role in supporting decision-making processes in public health.^17^ In England, a number of priority-setting tools have been developed over the years to facilitate decision-making processes in public health.^18–20^ However, there is little published empirical research on the ways in which prioritization decisions are reached within the new public health system.^13^ Understanding how public health priorities are determined in local government, and how priority-setting tools might support decision-making, remains unclear.

### What this study adds

This study sought to build on the earlier research findings, briefly noted above, in order to evaluate the impact of the new tool in regard to the ring-fenced public health budget. It assessed the value and impact of PHE’s PF tool in three early adopter local authorities. Further research could explore the value of the tool in aiding investment and disinvestment decisions and its impact on spending.

### Limitations of this study

Our study is confined to three early adopter sites. Therefore, the findings are not representative of all 152 LAs in England. At the same time, regardless of the sample size, no study would be wholly representative given the diversity evident among local authorities. Crucially, local circumstances and context will influence adoption of the PF tool.

## Conclusions

The issues the PF seeks to address will not go away and some mechanism which provides a forum for engaged and informed deliberation about priorities and does so in an open, transparent manner will be required. From our research, it appears that the PF offers such a mechanism and one that our three sites broadly welcomed. Despite the challenges identified, the tool proved itself to be sufficiently robust to be adopted more widely by local authorities and their public health teams.

## Notes

**G. Maniatopoulos,** Senior Research Associate

**D.J. Hunter,** Emeritus Professor of Health Policy and Management

**J. Gray,** Associate Professor of Health Economics
